# Circ_0005576 Exerts an Oncogenic Role in Cervical Cancer via miR-1305-Dependent Regulation of PAIP1

**DOI:** 10.1007/s43032-022-00925-y

**Published:** 2022-04-04

**Authors:** Yajing Wang, Fang Du, Zongyuan Xie, Junhao Lai, Yuanjie Li, Yongping Xu, Rui Tong

**Affiliations:** 1 Department of MRI, North China University of Science and Technology Affiliated Hospital, Tangshan, 063000 People’s Republic of China; 2 Department of Hematology and Oncology, No.988 Hospital of Joint Logistic Support Force of the Chinese People’s of Liberation Army, Zhengzhou, 450042 People’s Republic of China; 3 Department of General Surgery, 987Th Hospital of PLA, Baoji, 721004 People’s Republic of China; 4 Department of Four Wards of Gynecology, Cancer Hospital of China Medical University, Liaoning Cancer Hospital & Institute, Shenyang, 110042 Liaoning Province People’s Republic of China

**Keywords:** CC, circ_0005576, miR-1305, PAIP1

## Abstract

Cervical cancer (CC) is a leading cause of high morbidity and mortality in women worldwide. Circular RNAs (circRNAs) are considered to be essential regulators of various cancers, including CC. The purpose of this study was to investigate the role and mechanism of circ_0005576 in CC progression. The levels of circ_0005576, miR-1305, and poly(A)-binding protein-interacting protein 1 (PAIP1) were detected by quantitative real-time PCR (qRT-PCR) or western blot assay. The stability and location of circ_0005576 were determined by ribonuclease R (RNase R) assay and subcellular fractionation distribution assay, respectively. Cell proliferation was evaluated by CCK-8 assay, EDU incorporation assay, and colony formation assay. Cell migration and invasion were assessed by transwell assay. The interactions between miR-1305 and circ_0005576 or PAIP1 were validated by dual-luciferase reporter assay. The protein expression of cyclin D1, vimentin, and matrix metallopeptidase 9 (MMP9) was tested by western blot. Moreover, mice xenograft models were constructed to analyze tumor growth in vivo. Circ_0005576 and PAIP1 were upregulated, while miR-1305 was downregulated in CC tissues and cells. Circ_0005576 was a stable circRNA that was mainly distributed in the cytoplasm of cells. Knockdown of circ_0005576 suppressed the proliferation, migration, and invasion of CC cells, while the silence of miR-1305 facilitated the development of CC cells. Meanwhile, circ_0005576 could sponge miR-1305 to promote PAIP1 expression. Furthermore, PAIP1 overexpression relieved the influence of circ_0005576 silence on the growth of CC cells. Additionally, circ_0005576 silence hindered CC tumor growth in vivo. Circ_0005576 depletion suppressed tumor development in CC by regulating the miR-1305/PAIP1 axis, suggesting that circ_0005576 might be a potential biomarker for CC treatment.

**Supplementary Information:**

The online version contains supplementary material available at 10.1007/s43032-022-00925-y.

## Background

Cervical cancer (CC) is one of the commonest forms of malignancy affecting women globally [1]. Despite many important advances that have also taken place in the diagnosis and treatment of CC, the survival rates of advanced CC were disappointing [2, 3]. Meanwhile, the molecular mechanism behind the recurrence and metastasis of CC is largely unclear. Therefore, finding potential targets that affect CC progression is of great significance for developing effective treatment strategies for CC patients.

Circular RNAs (circRNAs) are a type of covalently closed non-coding RNA that emerge as crucial regulators in various cancers [4, 5], including CC [6]. Moreover, circRNAs may function as competing endogenous RNAs through the circRNA/miRNA/mRNA networks in the pathology of CC [7]. For example, circUBAP2 facilitated cell proliferation and metastasis in CC via miR-361-3p/SOX4 axis [8]. And Circ_0008285 could promote CC progression by regulating SOX4 mediated by miR-211-5p [9]. Nevertheless, the specific mechanism of circ_0005576 in CC tumorigenesis remains largely hidden. Ma et al. has proved that circ_0005576 was upregulated in CC and had stimulative effects on CC proliferation, migration, and invasion via miR-153-3p/KIF20A axis [10]. However, whether circ_0005576 could regulate CC development through other miRNAs and pathways is unclear. Thus, it is imperative to investigate the precise action and mechanism of circ_0005576 in the carcinogenesis of CC.

MicroRNAs (miRNAs) are small, non-coding RNA molecules that are associated with the progression of many cancers including CC, serving as tumor promoters or inhibitors [11, 12]. For instance, miR-96 served as a tumor promoter in proliferative, migratory and invasive abilities of CC cells by targeting CAV-1 [13]. While Kan et al. found that the miR-1294 level was declined and impeded cell viability and metastasis by interacting with FLOT1 [14]. Furthermore, miR-1305 was low expressed and blocked CC cells proliferation and metastasis via the Wnt/β-catenin pathway [15]. However, the correlation between circ_0005576 and miR-1305 in CC progression is still largely unclear.

Poly(A)-binding protein-interacting protein 1 (PAIP1) is a key modulator in the translation initiation of messenger RNA (mRNA) as a result of binding to PABP [16, 17]. PAIP1 could play pivotal roles in the development of multifarious diseases, including breast cancer [18], gastric cancer [19], and lung adenocarcinoma (LADC) [20]. Meanwhile, PAIP1 was upregulated and PAIP1 silence blocked cell progression in CC [21], while the evidence is still lacking about the specific pathogenic role of PAIP1 in CC.

In this study, the expression and function of circ_0005576 in CC development were illuminated. Furthermore, the correlation between circ_0005576 and miR-1305/PAIP1 pathway was elucidated. This finding might provide a therapeutic target for CC treatment.

## Materials and Methods

### Clinical specimens

Fifty-five paired CC tissues and adjacent para-carcinoma tissues were acquired from CC patients who underwent resection surgery at North China University of Science and Technology Affiliated Hospital. None of the enrolled patients accepted preoperative radiotherapy or chemotherapy. Written informed consent was signed by all participants before operation. This procedure was ratified by the Ethics Committee of North China University of Science and Technology Affiliated Hospital.

### Cell Culture

CC cell lines (HeLa and SiHa) and normal human cervical epithelial cell line HcerEpic were commercially acquired from BeNa Culture Collection (Suzhou, Jiangsu, China). All cells were cultured in Dulbecco’s modified Eagle’s medium (DMEM; Invitrogen, Carlsbad, CA, USA) supplemented with fetal bovine serum (FBS, 10%; Solarbio, Beijing, China) and streptomycin/penicillin (1%, Solarbio) at 37˚C with 5% CO_2_ in a moist atmosphere.

### Cell Transfection

The small interfering RNA (siRNA) specifically targeting circ_0005576 (si-circ_0005576) and corresponding negative control (si-NC), as well as miR-1305 mimic and inhibitor (miR-1305 and anti-miR-1305) and their matching negative controls (miR-NC and anti-NC) were obtained from GenePharma (Shanghai, China). The overexpression vector of PAIP1 (pcDNA-PAIP1) was constructed through cloning the full-length sequence of PAIP1 into the empty pcDNA3.1 vector (pcDNA) (Life Technologies, Grand Island, NY, USA), respectively. After reaching ~ 80% confluence, HeLa and SiHa cells were transfected with designated oligonucleotides or vectors using Lipofectamine 3000 reagent (Invitrogen).

### qRT-PCR

Total RNA extraction was accomplished via TRIzol reagent (Solarbio). Complementary DNA (cDNA) was synthesized with the PrimeScript RT Master Mix (Takara, Dalian, China). Subsequently, quantitative real-time polymerase chain reaction (qRT-PCR) was executed by using the CFX96 Real-time PCR Detection System (Bio-Rad, Hercules, CA, USA) with the SYBR Green PCR Master Mix (Bio-Rad). The relative expression of RNAs was calculated via the 2^−ΔΔCt^ method. The glyceraldehyde-3-phosphate dehydrogenase (GAPDH) or U6 was regarded as an internal control. The primers included the following: circ_0005576, F: 5′-TGCCAAGAACAAACAGAAGC-3′ and R: 5′-TTTTACCAACAGCACCATCG-3′; miR-1305, F: 5′-TTTTCAACTCTAATGGGAG-3′ and R: 5′-GAACATGTCTGCGTATCTC-3′; PAIP1, F: 5′-CAAATGGACAGGTTACAAGAGCAG-3′ and R: 5′-GCATCTTCCAAAACTGATCCTGTC-3′; GAPDH, F: 5′-GGGAAACTGTGGCGTGAT-3′ and R: 5′-GAGTGGGTGTCGCTGTTGA-3′; U6, F: 5′-CTCGCTTCGGCAGCACA-3′ and R: 5′-AACGCTTCACGAATTTGCGT-3′; 18S ribosomal RNA (rRNA), F: 5′-CAGCCACCCGAGATTGAGCA-3′ and R: 5′-TAGTAGCGACGGGCGGGTGT-3′.

### RNase R Digestion

To explore the stability of circ_0005576, an RNase R digestion assay was performed. Briefly, 2 μg RNA was incubated with or without RNase R (3 U/μg) (Epicentre, Madison, WI, USA) for 30 min at 37 °C. Subsequently, the levels of circ_0005576 and GAPDH were examined using qRT-PCR.

### Cellular Fractionation Distribution

The nuclear RNA and cytoplasmic RNA were extracted from CC cells using the PARIS Kit (Life Technologies). The level of circ_0005576 was examined using qRT-PCR. U6 or 18S rRNA was respectively served as the control of nucleus or cytoplasm in HeLa and SiHa cells.

### Cell Proliferation Assay

For the CCK-8 assay, transfected CC cells (1 × 10^3^ cells/well) were plated into 96-well plates and kept for 24 h, 48 h, or 72 h at 37 °C. Next, 10 μL of CCK-8 solution (Solarbio) was added to each well. After incubation for 2 h, the optical density of each well was recorded at a wavelength of 450 nm using a microplate reader (Bio-Rad).

### EDU Incorporation Assay

5-Ethynyl-2-deoxyuridine (EDU) incorporation assay was also implemented to detect cell proliferation. Briefly, transfected CC cells were plated into 96-well plates and then incubated with 50 μM EDU (Invitrogen) for 2 h at 37 °C. Then, the cells were fixed with 4% formaldehyde (Solarbio) for 15 min at room temperature (RT) after being washed with PBS three times. Then, 0.5% Triton X-100 was applied for 15 min at RT to permeabilize cells. Subsequently, the cells were incubated with a 1 × Apollo reaction cocktail for 30 min after washing with PBS three times. Finally, DNA was stained with 4′,6-diamidino-2-phenylindole (DAPI) for 30 min and the numbers of proliferative cells were analyzed under fluorescence microscopy (Olympus, Tokyo, Japan).

### Colony Formation Assay

For the colony formation assay, CC cells were maintained in six-well plates at a concentration of 5000 cells/well, and incubated at 37 °C for 14 days in a humidified atmosphere of 5% CO_2_. Subsequently, the colonies were stained with 1% crystal violet solution (Solarbio) after fixed with 4% paraformaldehyde (Solarbio). The visible colonies were photographed and counted.

### Transwell Migration and Invasion Assay

For cell migration assay, the treated cells were suspended in a serum-free culture medium and plated into the upper chamber with an 8-μm pore membrane filter (Costar, Cambridge, MA, USA). The lower chamber was replenished with a complete medium containing FBS (10%). After 24-h incubation, the cells on the lower surface were fixed with paraformaldehyde (4%, Solarbio) and then stained with 0.5% crystal violet (Solarbio). For the cell invasion test, the steps were the same as the migration assay, except for the coverage of Matrigel (BD Biosciences, San Diego, CA, USA) on the 8 μm pore membrane filter of the upper chamber. The cells were photographed and counted with a light microscope (Olympus) at 100 × magnification in three randomly selected fields.

### Western Blot Assay

After isolating the protein from tissues and cells with RIPA lysis buffer (Solarbio), the protein samples were quantified with the BCA Protein Assay Kit (Pierce, Rockford, IL, USA). Then, total protein was separated using sodium dodecyl sulfate–polyacrylamide gel electrophoresis (SDS-PAGE) and transferred onto polyvinylidene difluoride (PVDF) membranes (Millipore, Billerica, MA, USA). After being blocked with 5% non-fat milk for 2 h, the membranes were probed with primary antibodies against cyclin D1 (1:3000, ab226977; Abcam, Cambridge, UK), vimentin (ab137321, 1: 2000; Abcam), matrix metallopeptidase 9 (MMP9, ab76003, 1:2000; Abcam), PAIP1 (ab181359, 1: 2000; Abcam), or GAPDH (1:2500, ab9485; Abcam) at 4 °C overnight. GAPDH was deemed as a loading control. Thereafter, the membranes were incubated with the secondary antibody (1:20,000, ab205718; Abcam) at RT for 2 h. Finally, the immunoblots were visualized via enhanced chemiluminescence (ECL) solution (Beyotime, Shanghai, China).

### Flow Cytometry Assay

After being fixed with 70% cold ethanol, transfected CC cells were dyed using Propidium Iodide (PI, Selleck, Shanghai, China) at room temperature. Twenty minutes later, the flow cytometer was used to analyze the percentages of CC cells in G0-G1, S, and G2-M phases.

### Dual-Luciferase Reporter Assay

The fragment of wild-type circ_0005576 or PAIP1 3′UTR containing the miR-1305 binding sequence was inserted into the pmirGLO luciferase vector (Promega, Madison, WI, USA) to construct circ_0005576 wt or PAIP1 3′UTR wt reporter vector, respectively. The mutant luciferase reporter vector (circ_0005576 mut or PAIP1 3′UTR mut) was generated through mutating the base at the miR-1305 binding sequence. Then, the corresponding luciferase reporter vectors were introduced into CC cells together with miR-NC or miR-1305. The luciferase activities were analyzed via Dual-Lucy Assay Kit (Solarbio).

### RIP Assay

Furthermore, the binding between circ_0005576 and miR-1305 was verified using a Magna RIP TM RNA-Binding Protein Immunoprecipitation Kit (Millipore). In short, HeLa and SiHa cells were lysed in RNA immunoprecipitation (RIP) buffer, followed by incubation at 4 °C overnight with against Argonaute2 (Anti-Ago2; Abcam) or immunoglobulin G (anti-IgG; Abcam) before treating magnetic protein A/G beads for 2 h. At last, the enrichment of circ_0005576 and miR-1305 in the beads was analyzed using qRT-PCR.

### Xenograft Assay

Xenograft mice models were constructed to examine the effects of circ_0005576 on CC tumor growth in vivo. For stable knockdown of circ_0005576, lentivirus carrying short hairpin RNA targeting circ_0005576 (sh-circ_0005576: F: 5′-CACCACACAGGTCATCATCAGATTTCGAAAAATCTGATGATGACCTGTG-3′; R: 5′-AAAACACAGGTCATCATCAGATTTTTCGAAATCTGATGATGACCTGTGT-3′) and corresponding negative control (sh-NC) were purchased from GenePharma. Subsequently, HeLa cells (1 × 10^6^ cells/0.1 mL PBS) stably transfected with sh-circ_0005576 or sh-NC were subcutaneously injected into the right flanks of 12 BALB/c nude mice (4-week-old, Beijing Vital River Laboratory Animal Technology Co., Ltd., Beijing, China) (6 mice/group). Tumor volume was measured every 7 days with the equation of (length × width^2^)/2. Until day 35, the mice were sacrificed and the xenografts were excised and weighed. The xenograft tissues were then used for subsequent qRT-PCR and western blot analysis. The xenograft experiment was authorized by the Animal Ethics Committee of North China University of Science and Technology Affiliated Hospital.

### IHC Analysis

The paraffin-embedded tissue sections from transplanted mice were divided into 4 µm slices after dewaxing. After that, the slices were co-reacted with primary antibodies against Ki-67 (ab16667, 1:200; Abcam) at 4 °C overnight, followed by the addition of secondary antibody (1:5000, ab205718; Abcam) for 1 h in the indoor environment. Afterward, the slices were stained by 3,3′-diaminobenzidine solution (DAB; Beyotime), and the nuclei were counterstained with hematoxylin (Beyotime). Lastly, the slices were observed using a microscope (Olympus).

### Statistical Analysis

Data were displayed as mean ± standard deviation with 3 repeated experiments. The differences were assessed using Student’s *t* test or one-way analysis of variance through GraphPad Prism 7.0 software (GraphPad, San Diego, CA, USA). The linear relationship between miR-1305 and circ_0005576 or PAIP1 in CC tissues was assessed via Spearman’s correlation coefficient. *P* < 0.05 was deemed to be statistically significant.

## Results

### Circ_0005576 Was Highly Expressed in CC Tissues and Cells

**Fig. 1:**
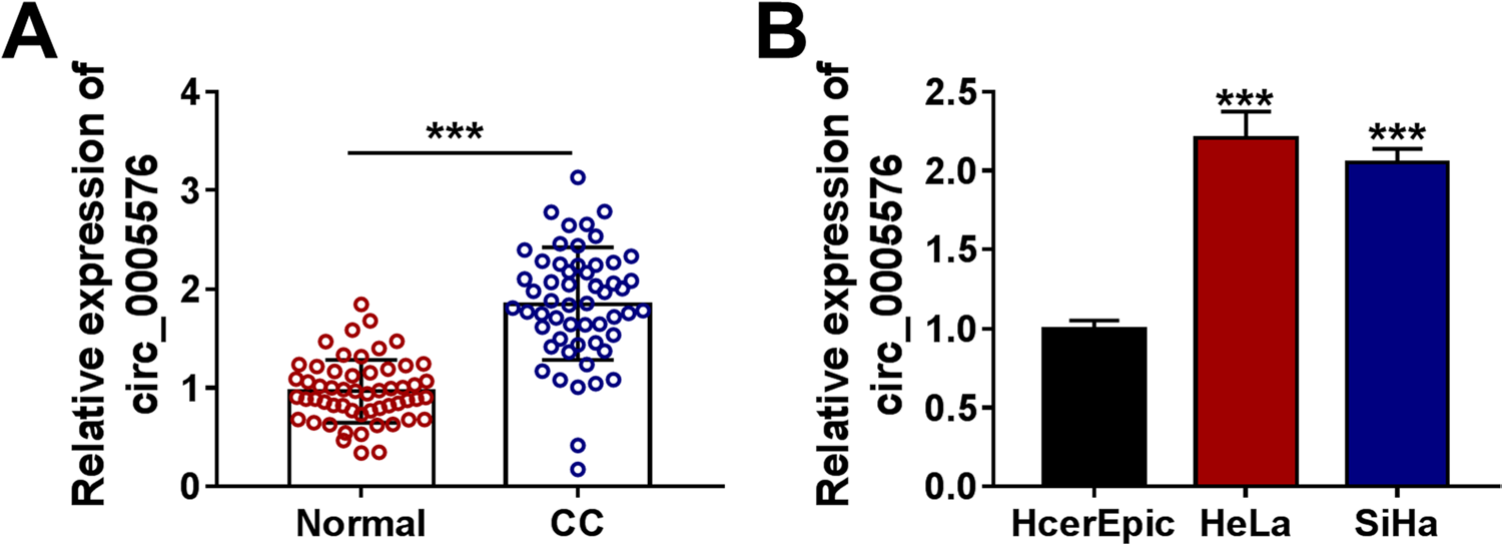
Circ_0005576 was upregulated in CC tissues and cells. **A** The expression of circ_0005576 was measured in CC tissues (*n* = 55) and matched normal tissues (*n* = 55) using qRT-PCR. **B** The expression of circ_0005576 in HeLa, SiHa, and normal HcerEpic cells was tested by qRT-PCR. ***P < 0.001

To explore the biological action of circ_0005576 in CC, the expression of circ_0005576 in 55 paired CC cancerous tissues and peritumor normal tissues were examined by qRT-PCR. The results manifested that circ_0005576 level was markedly elevated in CC tissues relative to that in matched normal tissues (Fig. 1A). Simultaneously, the level of circ_0005576 in HeLa and SiHa cells was significantly increased than that in normal HcerEpic cells (Fig. 1B). In order to detect the stability of circ_0005576, total RNAs extracted from HeLa and SiHa cells were treated with RNase R. As illustrated in Supplementary Fig. 1A and 1B, circ_0005576 was more resistant to RNase R digestion compared with the linear GAPDH mRNA. Meanwhile, the subcellular localization assay demonstrated that circ_0005576 was mainly located at the cytoplasm of HeLa and SiHa cells (Supplementary Fig. 1C and 1D). These data indicated that circ_0005576 was a stable transcript and was highly expressed in CC.

### Circ_0005576 Knockdown Inhibited the Proliferation, Migration, and Invasion in CC Cells

**Fig. 2:**
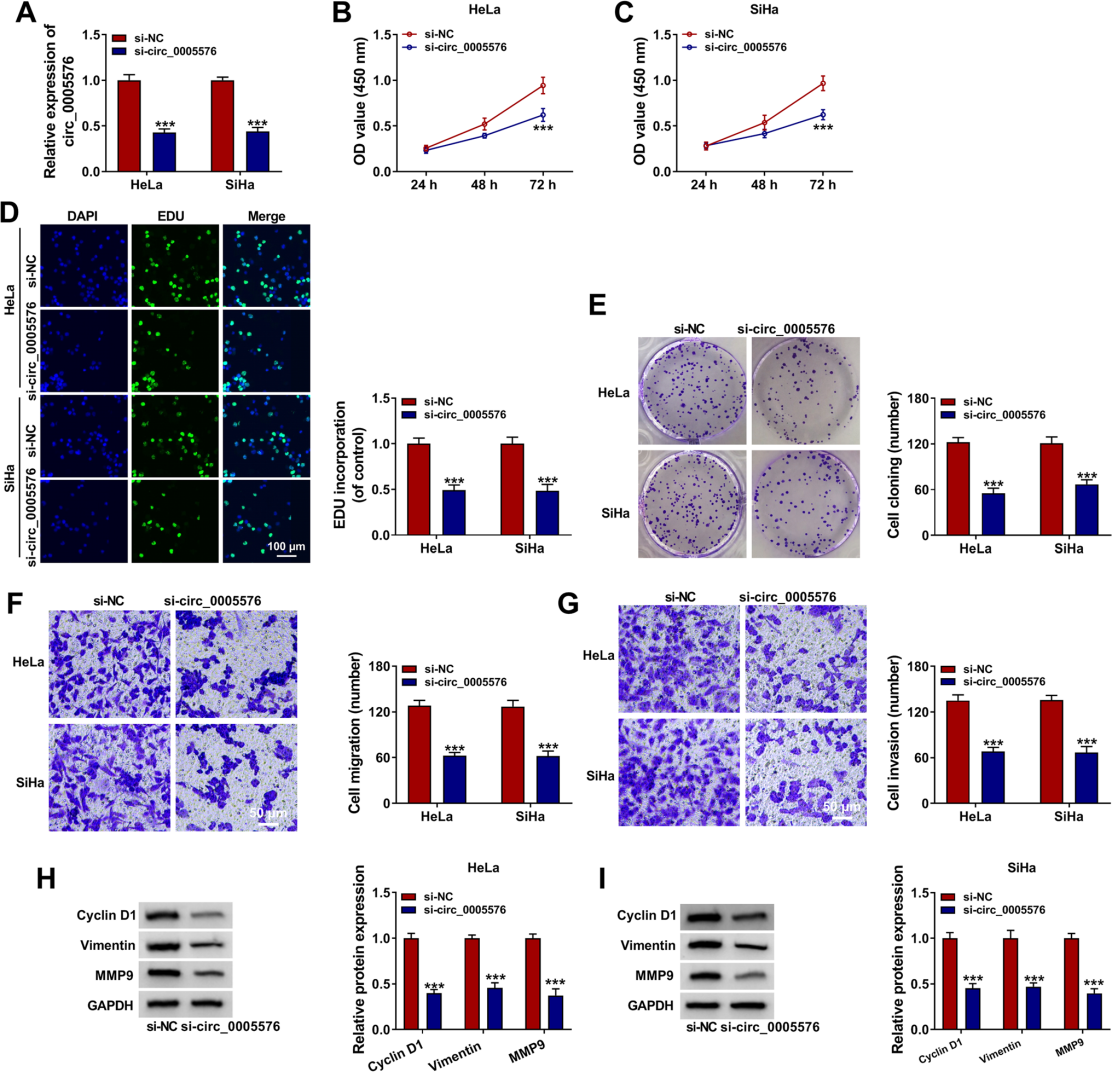
Circ_0005576 depletion inhibited the proliferation and metastasis in CC cells. HeLa and SiHa cells were transfected with si-NC or si-circ_0005576. **A** Circ_0005576 expression was determined by qRT-PCR. **B**–**D** The proliferation of HeLa and SiHa cells after transfection was assessed using CCK-8 assay and EDU incorporation assay. **E** The colony formation was detected with colony formation assay. **F**, **G** Cell migration and invasion were monitored by transwell assay. **H**, **I** The levels of cyclin D1, vimentin, and MMP9 were examined using western blot. ***P < 0.001

To investigate whether circ_0005576 served as a carcinogenic factor in CC development, knockdown experiments were performed. First, si-NC or si-circ_0005576 was introduced into HeLa and SiHa cells, and the knockdown efficiency of circ_0005576 was determined by qRT-PCR (Fig. 2A). CCK-8 assay illustrated that si-circ_0005576 transfection led to prominently declining proliferative ability in HeLa and SiHa cells (Fig. 2B and C). EDU incorporation assay testified that EDU incorporation was lessened in circ_0005576-depressed HeLa and SiHa cells (Fig. 2D), which again verified the catabatic proliferative ability of CC cells. Colony formation assay verified that transfection with si-circ_0005576 strikingly suppressed CC cell colony formation compared with the si-NC group (Fig. 2E). Furthermore, transwell assay presented that circ_0005576 knockdown remarkably impeded the migration and invasion of HeLa and SiHa cells (Fig. 2F and G). Besides, the levels of cell cycle-related protein cyclin D1 and metastasis-associated proteins vimentin and MMP9 in transfected HeLa and SiHa cells were markedly depressed (Fig. 2H and I). In addition, our data showed that HeLa and SiHa cell cycle progression was obviously repressed by circ_0005576 absence (Supplementary Fig. 2). Collectively, these data manifested that circ_0005576 depletion inhibited the malignant behaviors of CC cells.

### Circ_0005576 Served as a Sponge for miR-1305 in CC Cells

**Fig. 3:**
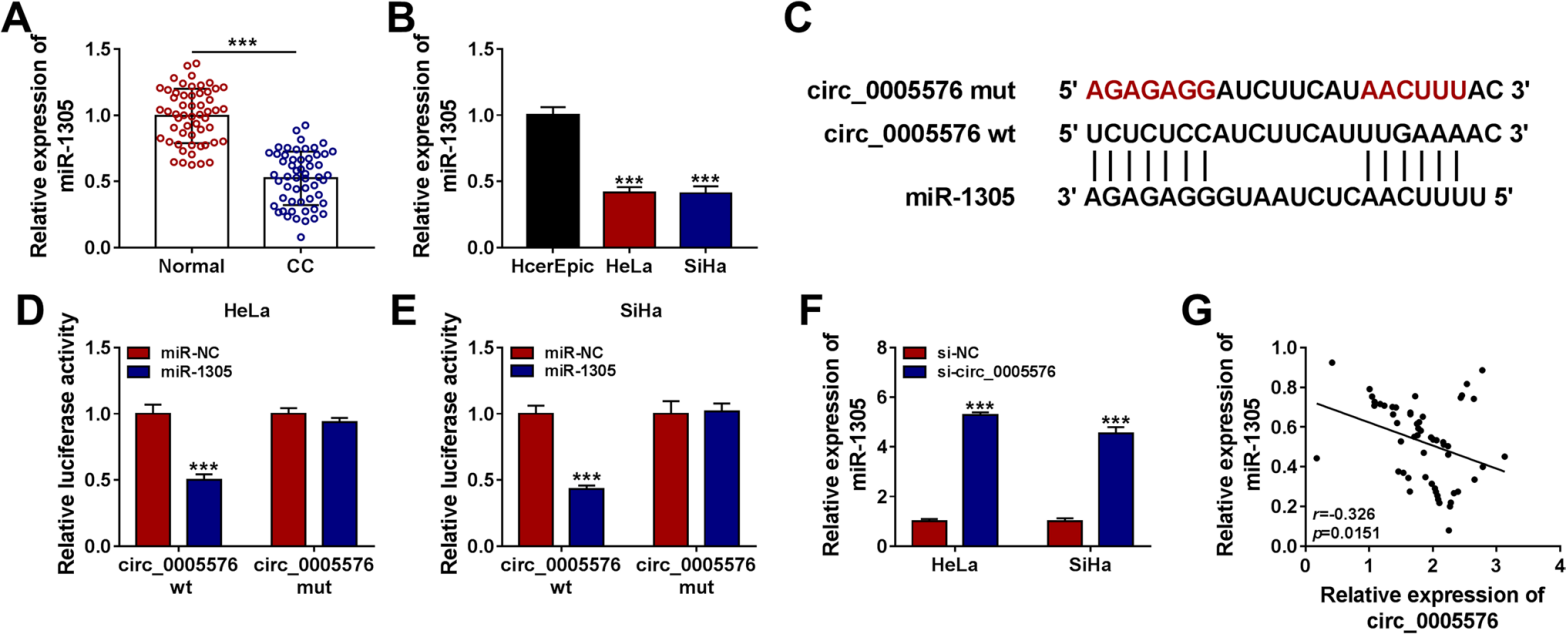
Circ_0005576 directly interacted with miR-1305 in CC cells. **A** The expression of miR-1305 was detected in CC tissues (*n* = 55) and matched normal tissues (*n* = 55) by qRT-PCR. **B** The level of miR-1305 in HeLa, SiHa, and HcerEpic cells was measured by qRT-PCR. **C** The putative binding sites for circ_0005576 and miR-1305 were predicted by Circular RNA Interactome database. **D**, **E** The luciferase activity was detected in HeLa and SiHa cells co-transfected with circ_0005576 wt or circ_0005576 mut and miR-1305 or miR-NC. **F** The level of miR-1305 was measured in HeLa and SiHa cells introduced with si-NC or si-circ_0005576. **G** The correlation between circ_0005576 and miR-1305 in CC tissues was analyzed using Spearman’s correlation coefficient. ***P < 0.001

Next, the molecular mechanism of circ_0005576 in CC was explored. The cytoplasmic distribution of circ_0005576 counseled that it might have a possibility to sponge miRNA. The expression of miR-1305 in CC tissues and cells was strikingly downregulated than that in adjacent normal tissues and cells (Fig. 3A and B). The biological database circular RNA Interactome database (https://circinteractome.nia.nih.gov/) predicted 6 different miRNA candidates (miR-326, miR-515-5p, miR-1305, miR-217, miR-874, and miR-146b-3p) that had targeted binding sites with circ_0005576. Then, we detected the expression of these different miRNAs in HeLa and SiHa cells after circ_0005576 knockdown (transfected with si-circ_0005576). The results suggested that miR-326, miR-515-5p, miR-1305, miR-217, and miR-874 were all upregulated while miR-146b-3p was downregulated, as shown in Supplementary Fig. 3A and B. Therein, the greatest change was present in miR-1305 expression after circ_0005576 knockdown; therefore, miR-1305 was selected as the target of circ_0005576 in our study for our subsequent experiments. Subsequently, the putative binding sites between circ_0005576 and miR-1305 were shown (Fig. 3C). To verify this binding relationship, the dual-luciferase reporter assay was conducted. The results showed that miR-1305 mimic overtly reduced the luciferase intensity of circ_0005576 wt reporter relative to the control group, but did not affect that of circ_0005576 mut reporter (Fig. 3D and E). In parallel, RIP assay displayed that circ_0005576 and miR-1305 were enriched in Anti-Ago2 immunoprecipitates in HeLa and SiHa cells relative to the anti-IgG control groups (Supplementary Fig. 4). Moreover, the knockdown of circ_0005576 could expedite the expression of miR-1305 in CC cells (Fig. 3F). Subsequently, Spearman’s correlation analysis displayed that the expression of circ_0005576 and miR-1305 was negatively correlated in CC tissues (Fig. 3G). These results unveiled that circ_0005576 directly interacted with miR-1305 in CC.

### Inhibition of miR-1305 Accelerated the Progression of CC Cells

**Fig. 4:**
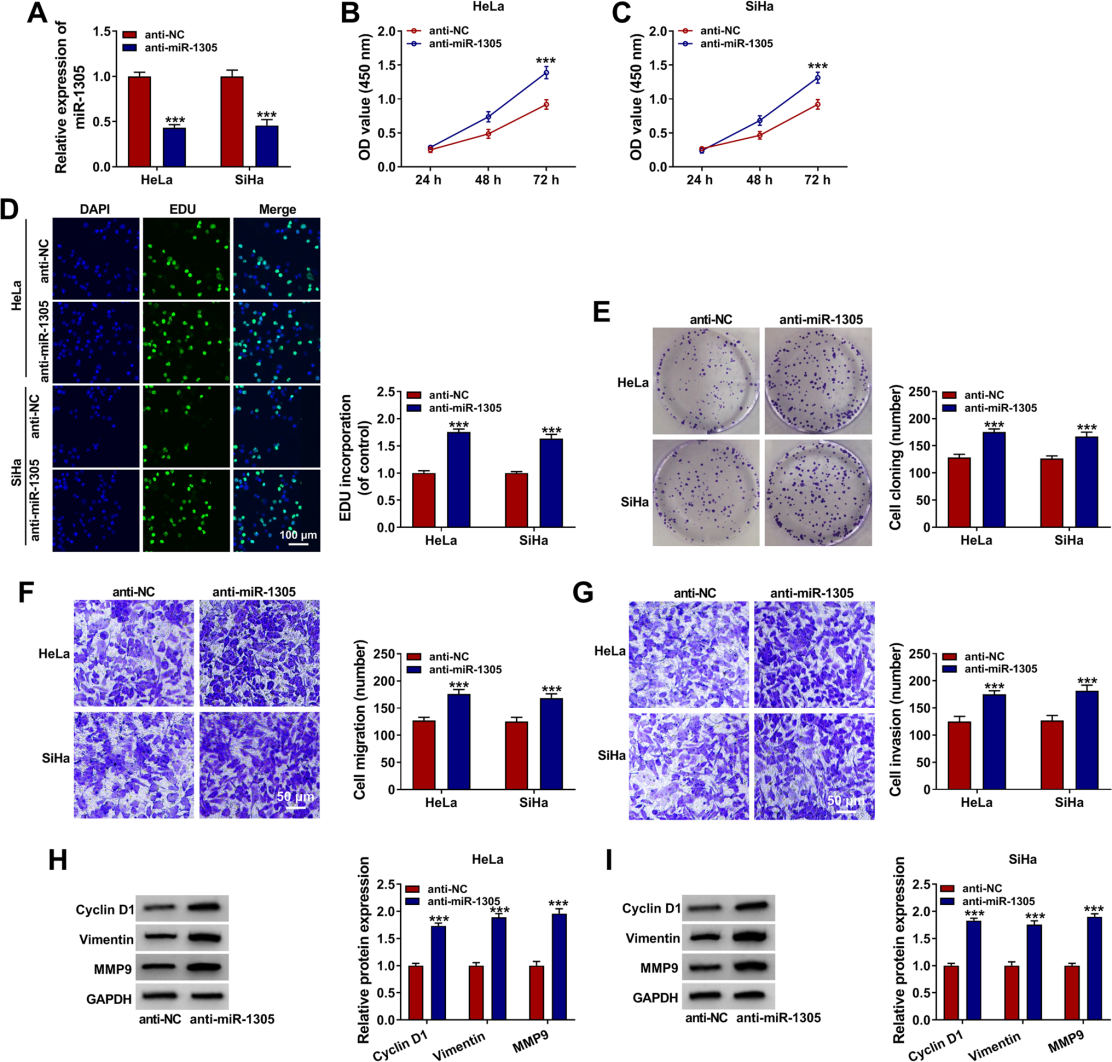
Silence of miR-1305 suppressed the progression of CC cells. HeLa and SiHa cells were introduced with anti-NC or anti-miR-1305. **A** The knockdown efficiency of anti-miR-1305 was measured by qRT-PCR in transfected HeLa and SiHa cells. **B**–**E** CCK-8 assay, EDU incorporation assay, and colony formation assay were conducted to evaluate cell proliferative capacity. **F**, **G** Transwell assay detected cell migration and invasion. **H**, **I** The levels of cyclin D1, vimentin and MMP9 were tested by western blot. ***P < 0.001

**Fig. 5:**
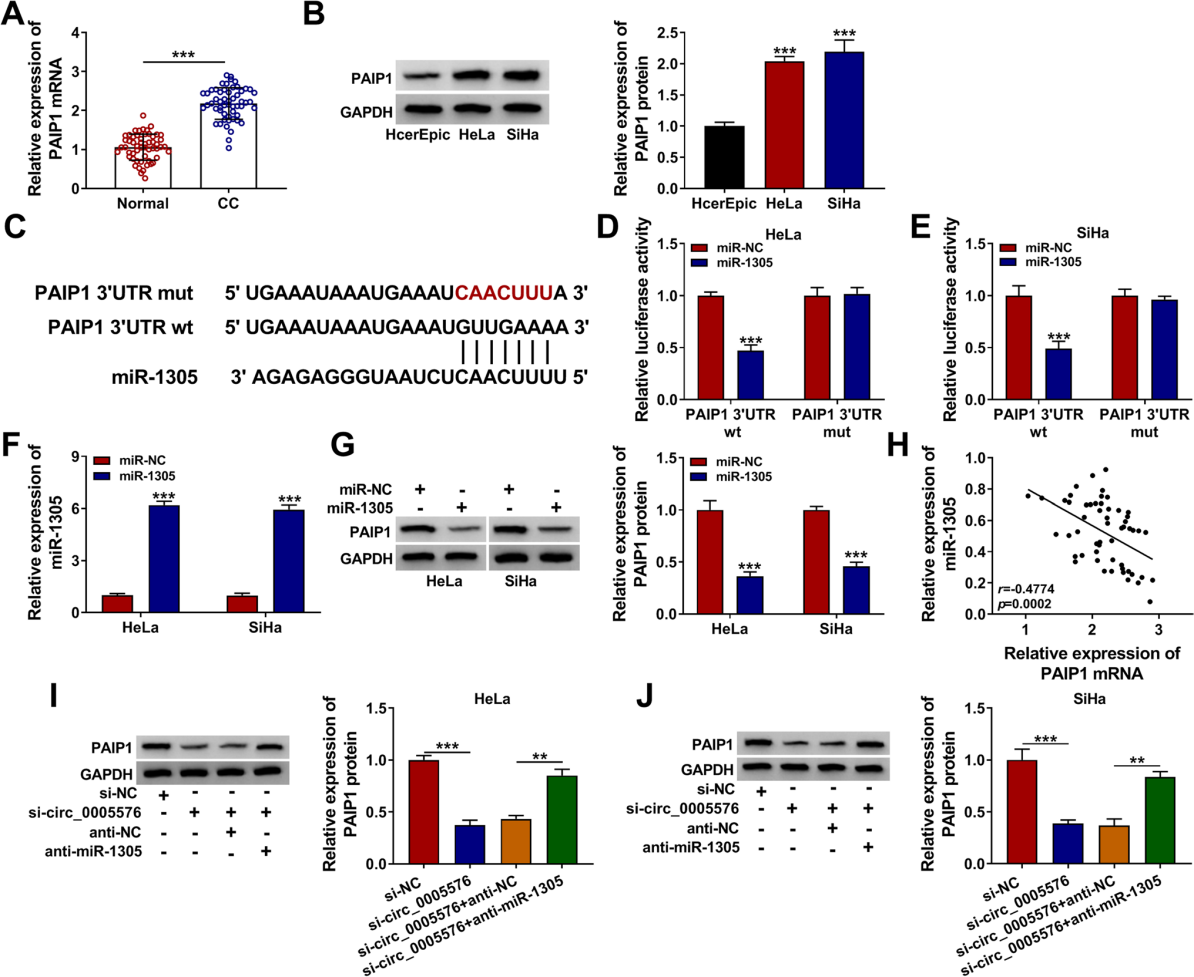
Circ_0005576 facilitated PAIP1 expression by sponging miR-1305. **A** The mRNA level of PAIP1 in CC tissues and normal tissues was measured by qRT-PCR. **B** The protein level of PAIP1 in CC cells and normal cells was detected by qRT-PCR. **C** The putative binding sites for miR-1305 and PAIP1 3′UTR were predicted by TargetScanHuman database. **D**, E The relationship between miR-1305 and PAIP1 was confirmed by dual-luciferase reporter assay. **F** The overexpression efficiency of miR-1305 was validated by qRT-PCR in HeLa and SiHa cells. **G** The protein level of PAIP1 was detected in HeLa and SiHa cells transfected with miR-1305 or miR-NC. **H** The correlation between miR-1305 and PAIP1 was analyzed by Spearman’s correlation analysis. **I**, **J** The expression of PAIP1 in HeLa and SiHa cells introduced with si-NC, si-circ_0005576, si-circ_0005576 + anti-NC, or si-circ_0005576 + anti-miR-1305 was examined by western blot, respectively. **P < 0.01, ***P < 0.001

To illuminate whether miR-1305 could take part in CC cell progression, miR-1305 was knocked down in HeLa and SiHa cells. First, the knockdown efficiency of miR-1305 was determined in HeLa and SiHa cells introduced with anti-NC or anti-miR-1305 using qRT-PCR (Fig. 4A). Then, the effect of miR-1305 on CC proliferation and metastasis was investigated. CCK-8 analysis illustrated that downregulation of miR-1305 dramatically augmented cell multiplication capacity (Fig. 4B and C). EDU incorporation assay further demonstrated the accelerated effect of anti-miR-1305 on cell proliferation (Fig. 4D). Moreover, colony formation assay demonstrated that suppression of miR-1305 signally enhanced cell clone formation (Fig. 4E). Additionally, transwell assay depicted that miR-1305 silence aggrandized the migration and invasion abilities of CC cells (Fig. 4F and G). Correspondingly, the expression of cyclin D1, vimentin, and MMP9 in transfected HeLa and SiHa cells was markedly fortified (Fig. 5H and I). Thus, these results evidenced that miR-1305 impeded cell development in CC.

### Circ_0005576 Facilitated PAIP1 Expression by Targeting miR-1305

To further explore the downstream mechanism of miR-1305 in CC development, the targeted gene of miR-1305 was sought. As depicted in Fig. 5A and B, the mRNA and protein levels of PAIP1 were remarkably elevated in CC tissues and cells. TargetScanHuman database (http://www.targetscan.org/vert_72/) predicted that PAIP1 3’UTR had the possible shared sequence with miR-1305 (Fig. 5C). Dual-luciferase reporter assay showed that enhanced miR-1305 expression overtly reduced the luciferase activity of PAIP1 3′UTR wt reporter but not that of PAIP1 3′UTR mut reporter in HeLa and SiHa cells (Fig. 5D and E), which confirming that PAIP1 was a downstream target of miR-1305. Then, the effect of miR-1305 on PAIP1 expression was explored. The overexpression efficiency of miR-1305 was verified by qRT-PCR (Fig. 5F). Furthermore, PAIP1 was remarkably decreased in CC cells introduced with miR-1305 mimic (Fig. 5G). Furthermore, the expression of PAIP1 was negatively correlated with miR-1305 expression in CC tissues by Spearman’s correlation coefficient analysis (Fig. 5H). To clarify the influence of circ_0005576 in PAIP1 expression, HeLa and SiHa cells were transfected with si-NC, si-circ_0005576, si-circ_0005576 + anti-NC or si-circ_0005576 + anti-miR-1305. The result of western blot showed that circ_0005576 knockdown markedly reduced the protein level of PAIP1, while the effect was abolished after co-transfection with si-circ_0005576 and miR-1305 inhibitor (Fig. 5I and J). Altogether, these data reflected that circ_0005576 could sponge miR-1305 to elevate PAIP1 expression in CC.

### PAIP1 Overexpression Abrogated the Influence of circ_0005576 Silence on the Growth of CC Cells

**Fig. 6:**
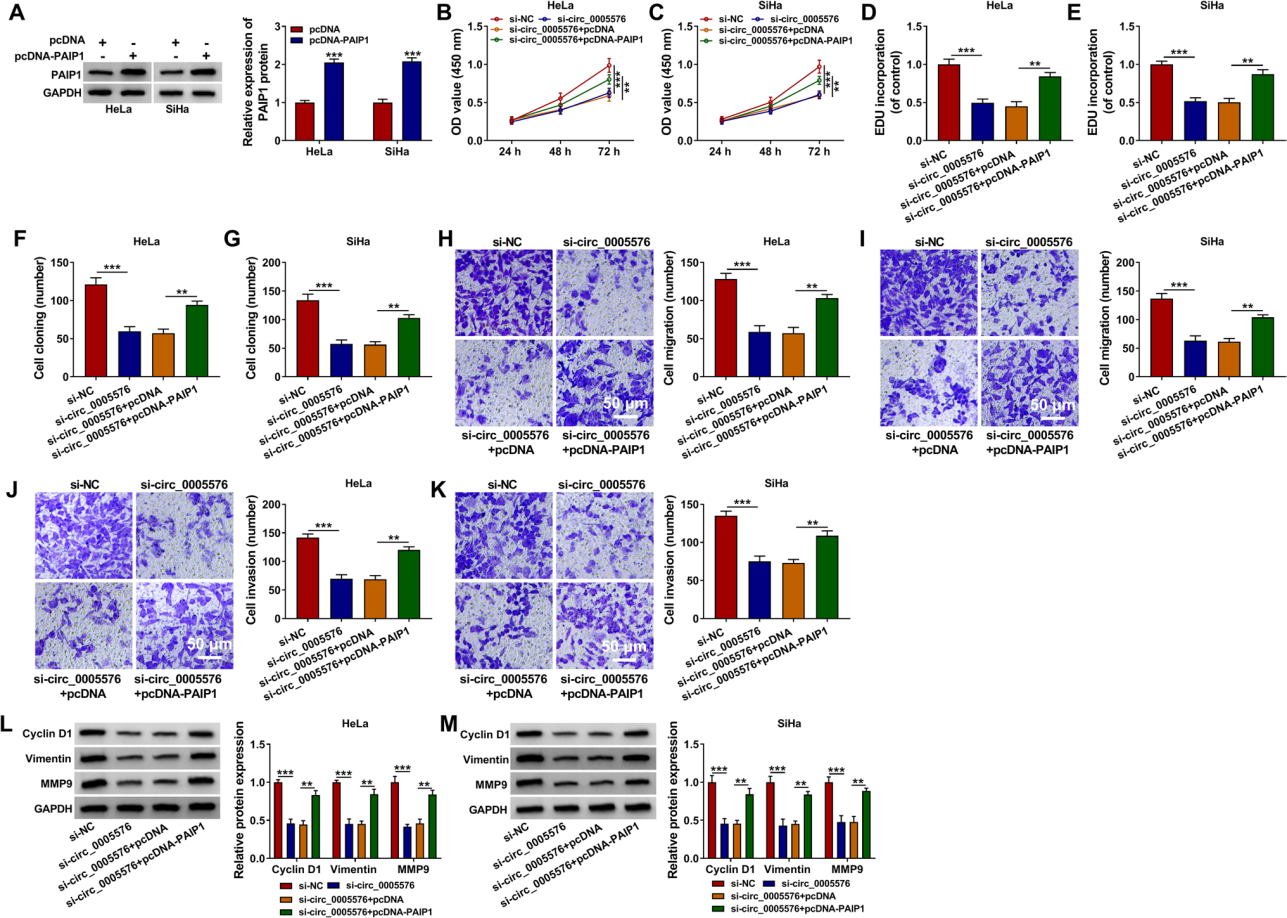
PAIP1 upregulation attenuated the effect of circ_0005576 silence on CC cell progression. **A** The transfection efficiency of pcDNA-PAIP1 was determined by qRT-PCR. **B**–**M** HeLa and SiHa cells were transfected with si-NC, si-circ_0005576, si-circ_0005576 + pcDNA, or si-circ_0005576 + pcDNA-PAIP1. **B**–**G** The cell proliferative capacity was tested by CCK-8 assay, EDU incorporation assay and colony formation assay. **H**–**K** The migration and invasion were evaluated by transwell assay. (L and M) The levels of cyclin D1, vimentin, and MMP9 were estimated by western blot. **P < 0.01, ***P < 0.001

Based on the above results, whether circ_0005576 could affect CC cell progression via regulating PAIP1 was further explored through a series of rescue experiments. Firstly, qRT-PCR was utilized to determine transfection efficiency of pcDNA-PAIP1, and the results illustrated that PAIP1 protein level in HeLa and SiHa cells after PAIP1 introduction was visibly elevated (Fig. 6A). For exploring the effects of circ_0005576 and PAIP1 on CC cell growth, HeLa and SiHa cells were transduced with si-NC, si-circ_0005576, si-circ_0005576 + pcDNA, or si-circ_0005576 + pcDNA-PAIP1, respectively. As displayed in Fig. 6B–G, overexpression of PAIP1 reversed the suppressive impacts of circ_0005576 silence on cell proliferative ability. Meanwhile, PAIP1 augmentation overturned si-circ_0005576-mediated repressive influence on cell metastatic capacities (Fig. 6H–K). Additionally, the lessened expression of cyclin D1, vimentin, and MMP9 was also relieved by PAIP1 addition in circ_0005576-declined CC cells (Fig. 6L and M). Consequently, these data attested that circ_0005576 knockdown could hinder CC cell progression via decreasing PAIP1 expression.

### Circ_0005576 Silence Inhibited CC Tumor Growth *In vivo*

**Fig. 7:**
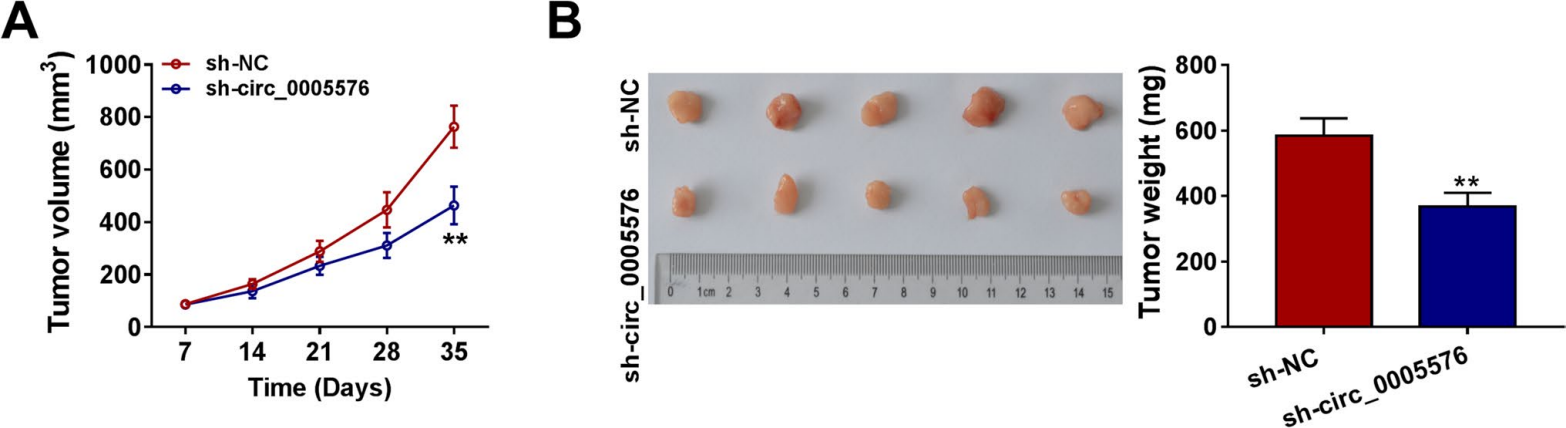
Circ_0005576 knockdown impeded CC tumor growth in vivo. HeLa cells transfected with sh-NC or sh-circ_0005576 were subcutaneously injected into the nude mice. **A** The volumes of the xenografts were measured once a week. **B** After 5 weeks, the mice were killed and the xenograft tumors were weighed. **P < 0.01

Considering that circ_0005576 knockdown could block the malignancy of CC cells in vitro, the xenograft model was constructed to further explore the role of circ_0005576 in tumorigenesis in vivo. The nude mice were inoculated with HeLa cells transfected with sh-NC or sh-circ_0005576. The results revealed that tumor volume and weight in the sh-circ_0005576 group were all overtly reduced relative to those of the sh-NC group (Fig. 7A and B). QRT-PCR assay confirmed that the abundance of circ_0005576 was reduced while miR-1305 level was raised in circ_0005576-silenced mice tumor tissues (Supplementary Fig. 5A). Concurrently, the protein level of PAIP1 was decreased in the sh-circ_0005576 group (Supplementary Fig. 5B). Also, western blot assay exhibited that circ_0005576 absence was able to apparently repress the expression of vimentin and MMP9 in the mice tumor tissues (Supplementary Fig. 5C). Likewise, immunohistochemistry (IHC) analysis presented that the expression level of Ki-67 was suppressed by the downregulation of circ_0005576 (Supplementary Fig. 5D). Taken together, these data confirmed that knockdown of circ_0005576 hindered CC tumor growth in vivo.

## Discussion

The deregulated expression of circRNAs and miRNAs has been reported to participate in the pathogenic processes of CC tumorigenesis [22]. However, the specific molecular mechanism of circ_0005576 in CC is still indistinct. This study explored the role of circ_0005576 and the potential mechanism of circRNA/mRNA/mRNA network in CC etiology.

A crowd of studies indicates that plentiful circRNAs are differentially expressed in tumor tissues and may be related to the carcinogenesis of cancers [23, 24]. Meanwhile, many circRNAs are implicated in the tumorigenesis of CC and may serve as potential biomarkers [6]. For instance, circNRIP1 accelerated cell migratory and invasive abilities in CC via targeting miR-629-3p [25]. And circ9119 could serve as an oncogene and facilitate cell proliferation via miR-126/MDM4 pathway in CC [26]. Nevertheless, the role of circ_0005576 is not fully expounded in CC. Circ_0005576 has been demonstrated to be overexpressed in CRC and was associated with tumor malignant progression through the miR-874/CDK8 axis in CRC [27]. Similarly, circ_0005576 was distinctly upregulated in CC tissues and cells, and circ_0005576 silence restrained CC cell proliferation and metastasis via the miR-153/KIF20A axis [10]. Nonetheless, whether there are other pathways mediated by circ_0005576 in the malignant phenotypes of CC is not entirely clear. Herein, we explored the role and regulatory mechanism of circ_0005576 in CC. In keeping with the above results, this study confirmed that circ_0005576 had enhanced expression in CC tissues and cells and might accelerate cell proliferation, migration and invasion of CC cells. In addition, we proposed that circ_0005576 silence also reduced CC tumor growth in vivo. All these data hinted that circ_0005576 was related to the carcinogenesis of CC and might be a latent biomarker for CC therapy.

Increasing evidence has discovered that miRNAs play significant roles in the development of CC by affecting specific pathways or via miRNA-mRNA network [11, 28]. Zhang et al. disclosed that miR-873-5p inhibited cell proliferation and metastasis by targeting TUSC3 in CC [29]. Furthermore, miR-1305 had been attested to block bladder cancer progression via Tgf-β2/smad3 pathway [30] and non-small cell lung cancer (NSCLC) progression via sponging MDM2 [31]. Besides, miR-1305 level was declined and miR-1305 inhibited cell proliferative and transferred capacities in CC via the Wnt/β-catenin pathway [15]. Here, this research revealed that miR-1305 was dramatically downregulated in CC and was targeted by circ_0005576. Meanwhile, the miR-1305 level was elevated by circ_0005576 silence. In addition, Spearman’s correlation coefficient analysis affirmed that there was a reverse correlation between circ_0005576 and miR-1305 expression. Thereby, it was deduced that circ_0005576 could reversely regulate miR-1305 expression through absorbing miR-1305. Moreover, the silence of miR-1305 could expedite cell proliferation and transferability in CC. All these results suggested that miR-1305 also took part in CC carcinogenesis which might be pertinent with circ_0005576. Then, the downstream mechanism of circ_0005576 and miR-1305 was further explored. The bioinformatics software was utilized to predict possible targets for miR-1305 in CC and PAIP1 was selected as a candidate gene.

Numerous studies have shown that PAIP1 was upregulated and was associated with the development of multifarious cancers. For example, Kim et al. found that PAIP1 expression was elevated in hepatocellular carcinoma and high expression of PAIP1 was associated with tumor invasion and worse five-year overall survival (OS) [32]. Guan et al. presented that PAIP1 was overexpressed in pancreatic cancer tissues, and depletion of PAIP1 signally lessened cell proliferation, metastasis and angiogenesis [33]. Additionally, PAIP1 was highly expressed and PAIP1 silence blocked cell growth in CC [21]. Consistent with this result, the current study certified that PAIP1 was notably upregulated in CC tissues and cells. Furthermore, PAIP1 was predicted to be a target of miR-1305, and the expression of PAIP1 was reversely regulated by miR-1305. Besides, it was verified that circ_0005576 could target miR-1305 to enhance PAIP1 expression in CC. More importantly, the rescue experiments indicated that PAIP1 overexpression could counteract the inhibitory effect of circ_0005576 downregulation on the malignant behaviors of CC cells. Overall, these findings gave evidence that circ_0005576 could participate in CC tumor occurrence and development via the miR-1305/PAIP1 axis.

In conclusion, this study unveiled that circ_0005576 triggered the proliferation, migration and invasion of CC cells through targeting miR-1305 to upregulate PAIP1 expression. These findings hinted that circ_0005576 might be a novel therapeutic marker for CC and this study also provided a latent therapeutic strategy for CC remedy.

## Supplementary Information

Below is the link to the electronic supplementary material.

Fig. S1 The stability and location of circ_0005576 in HeLa and SiHa cells. (A and B) The levels of circ_0005576 and GAPDH in HeLa and SiHa cells after RNase R treatment were evaluated with qRT-PCR. (C and D) The levels of circ_0005576, U6 and 18S rRNA in the cytoplasm and nucleus of HeLa and SiHa cells were assessed by qRT-PCR. ***P < 0.001.(PNG 59 kb)

High Resolution (TIF 307 kb)

Fig. S2 The effect of circ_0005576 knockdown on cell cycle arrest of CC cells. (A and B) Flow cytometry analysis of cell cycle arrest in HeLa and SiHa cells transfected with si-circ_0005576 or si-NC. *P < 0.05.(PNG 570 kb)

High Resolution (TIF 1191 kb)

Fig. S3 The relative expression of miRNAs in HeLa and SiHa cells after circ_0005576 knockdown. (A and B) The expression levels of different miRNAs in HeLa and SiHa cells transfected with si-circ_0005576 or si-NC were detected by qRT-PCR. *P < 0.05, **P < 0.01, ***P < 0.001.(PNG 103 kb)

High Resolution (TIF 373 kb)

Fig. S4 The binding between circ_0005576 and miR-1305 in CC cells. (A and B) RIP assay was used to verify the binding between circ_0005576 and miR-1305 in HeLa and SiHa cells. *P <  0.05. (PNG 46 kb)

High Resolution (TIF 236 kb)

Fig. S5 The functional role of circ_0005576 silencing in vivo. (A) The levels of circ_0005576 and miR-1305 were examined by qRT-PCR. (B) The protein level of PAIP1 was detected by western blot. (C) The protein levels of Vimentin and MMP9 were examined by western blot. (D) The Ki-67-positive cells in tumor tissues were detected by IHC analysis. ***P < 0.001. (PNG 615 kb)

High Resolution (TIF 1037 kb)
