# Supplementary figures and images for: Circ_0005576 Exerts an Oncogenic Role in Cervical Cancer via miR-1305-Dependent Regulation of PAIP1
Source: Reprod Sci. 2022 Apr 4;29(9):2647–58. doi: 10.1007/s43032-022-00925-y (PMC9444835; doi:10.1007/s43032-022-00925-y)

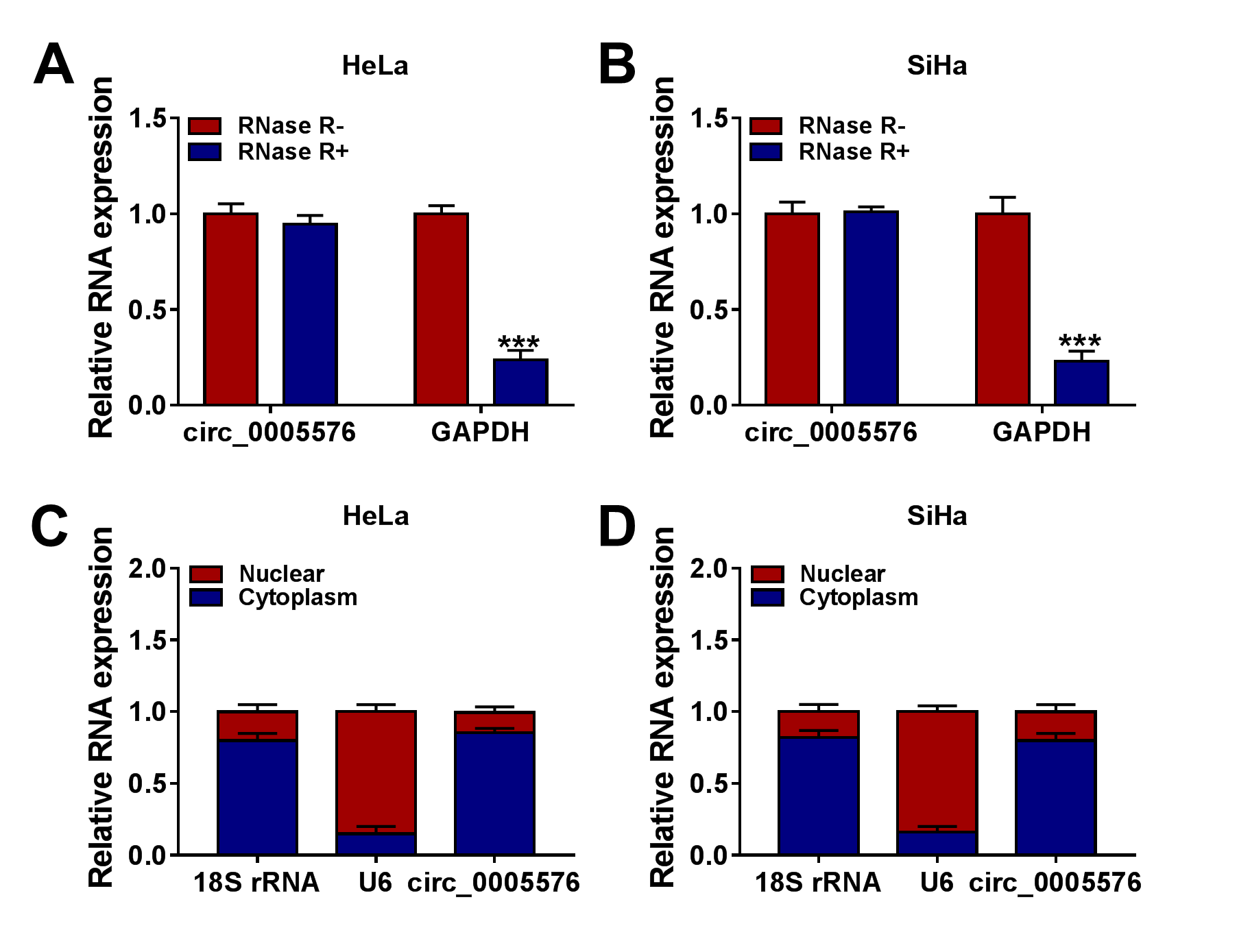

Supplement: Supplementary file 1 — The stability and location of circ_0005576 in HeLa and SiHa cells. (A and B) The levels of circ_0005576 and GAPDH in HeLa and SiHa cells after RNase R treatment were evaluated with qRT-PCR. (C and D) The levels of circ_0005576, U6 and 18S rRNA in the cytoplasm and nucleus of HeLa and SiHa cells were assessed by qRT-PCR. ***P < 0.001.(PNG 59 kb) [file 43032_2022_925_Fig8_ESM.png]

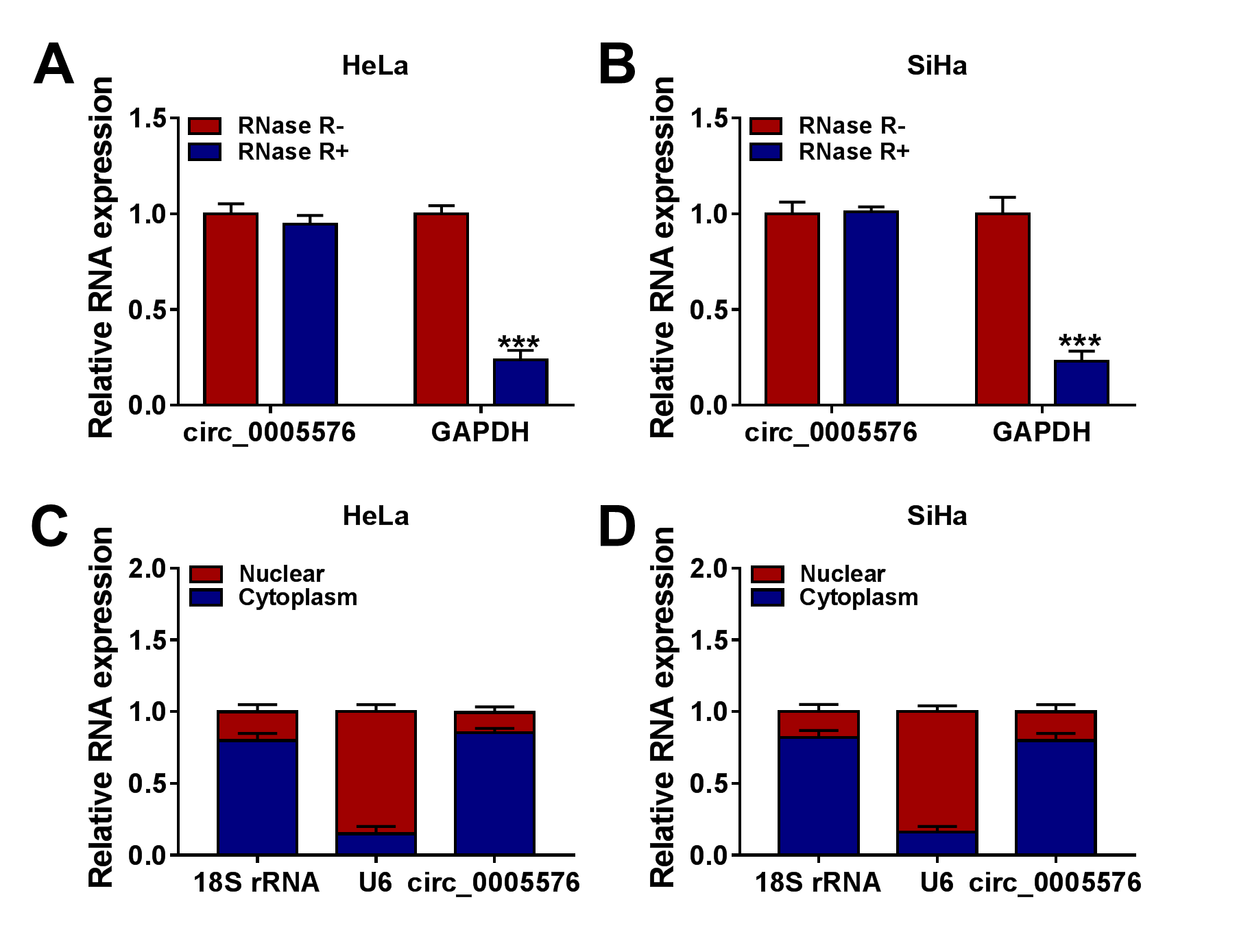

Supplement: Supplementary file 2 — High Resolution (TIF 307 kb) [file 43032_2022_925_MOESM1_ESM.tif]

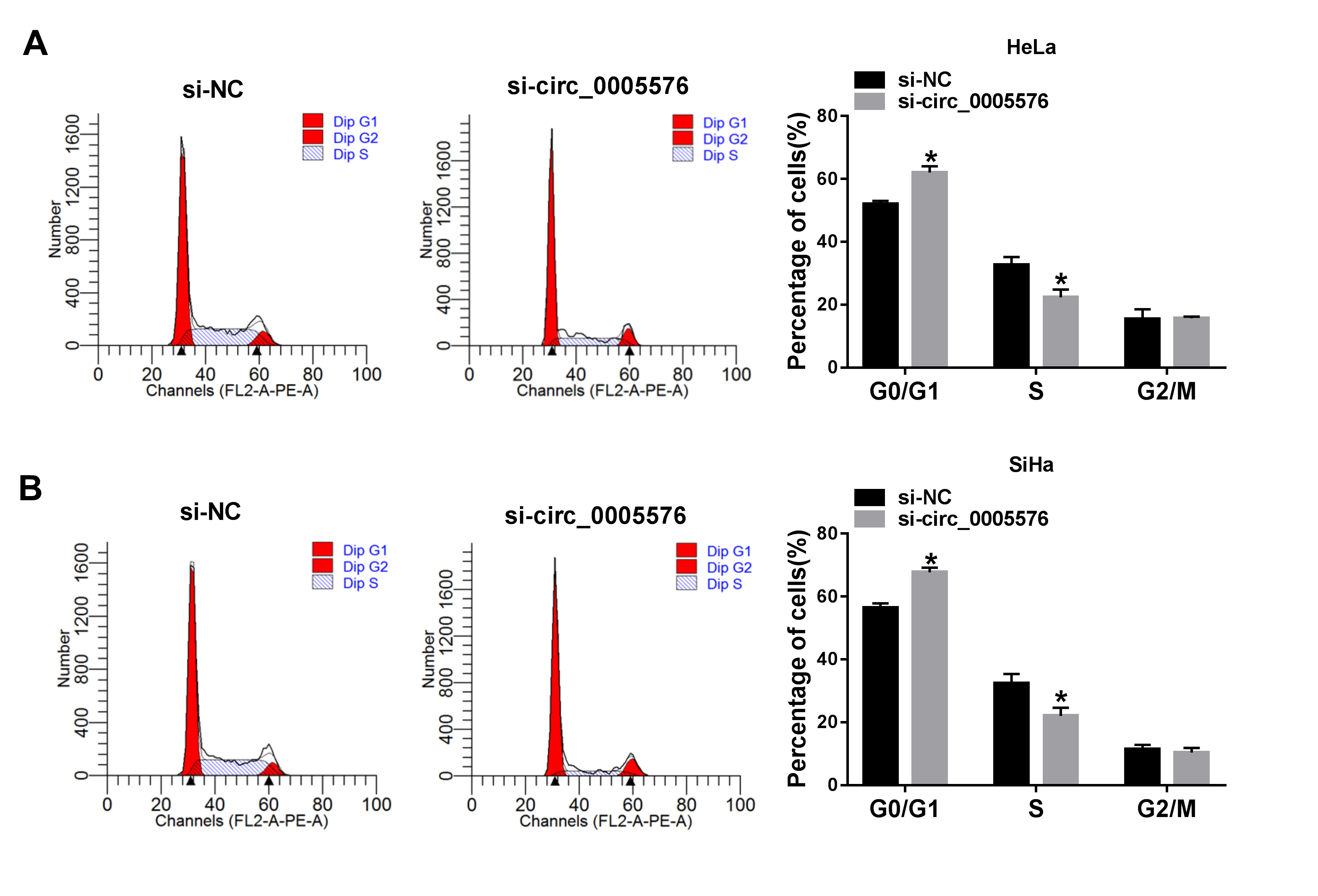

Supplement: Supplementary file 3 — The effect of circ_0005576 knockdown on cell cycle arrest of CC cells. (A and B) Flow cytometry analysis of cell cycle arrest in HeLa and SiHa cells transfected with si-circ_0005576 or si-NC. *P < 0.05.(PNG 570 kb) [file 43032_2022_925_Fig9_ESM.png]

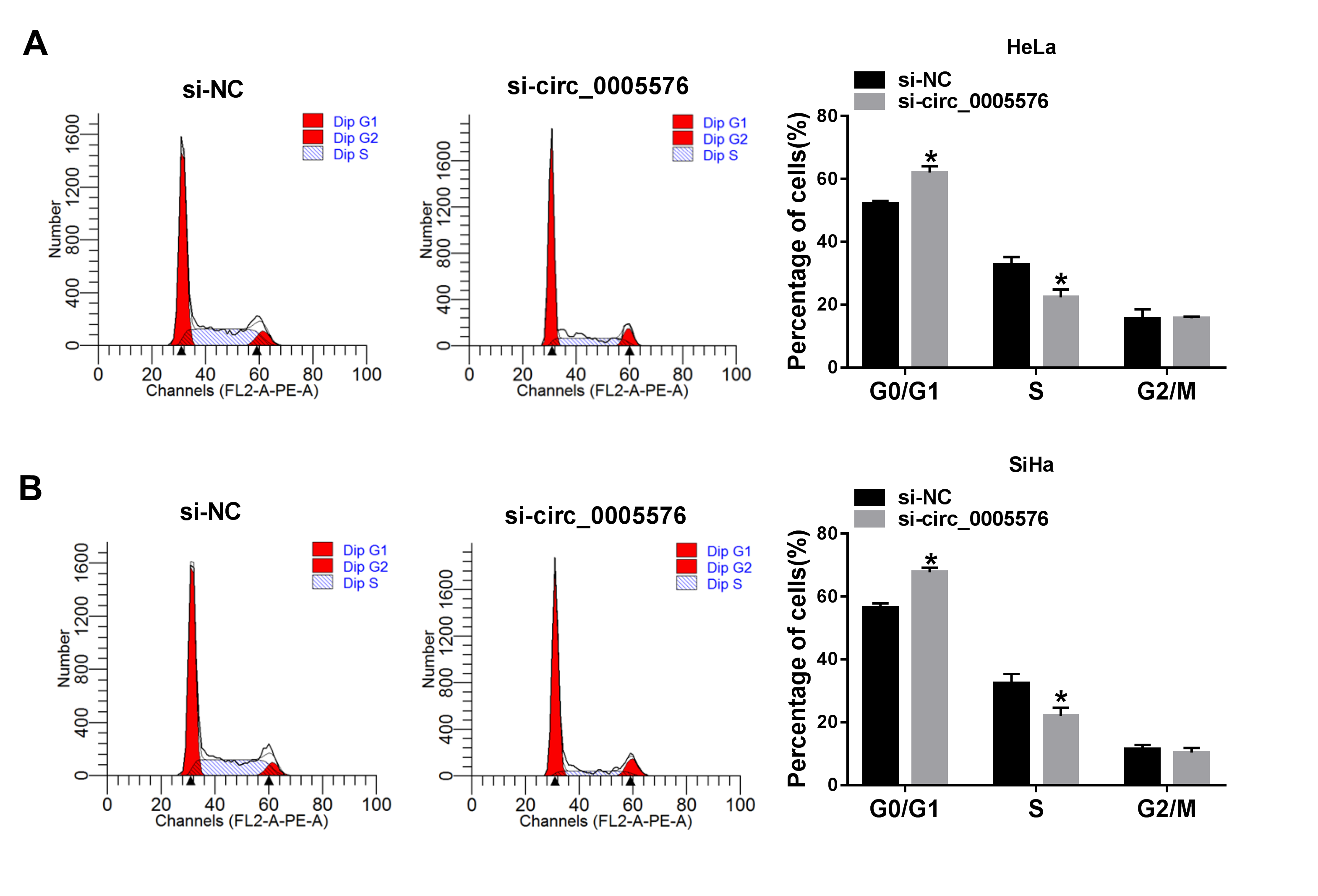

Supplement: Supplementary file 4 — High Resolution (TIF 1191 kb) [file 43032_2022_925_MOESM2_ESM.tif]

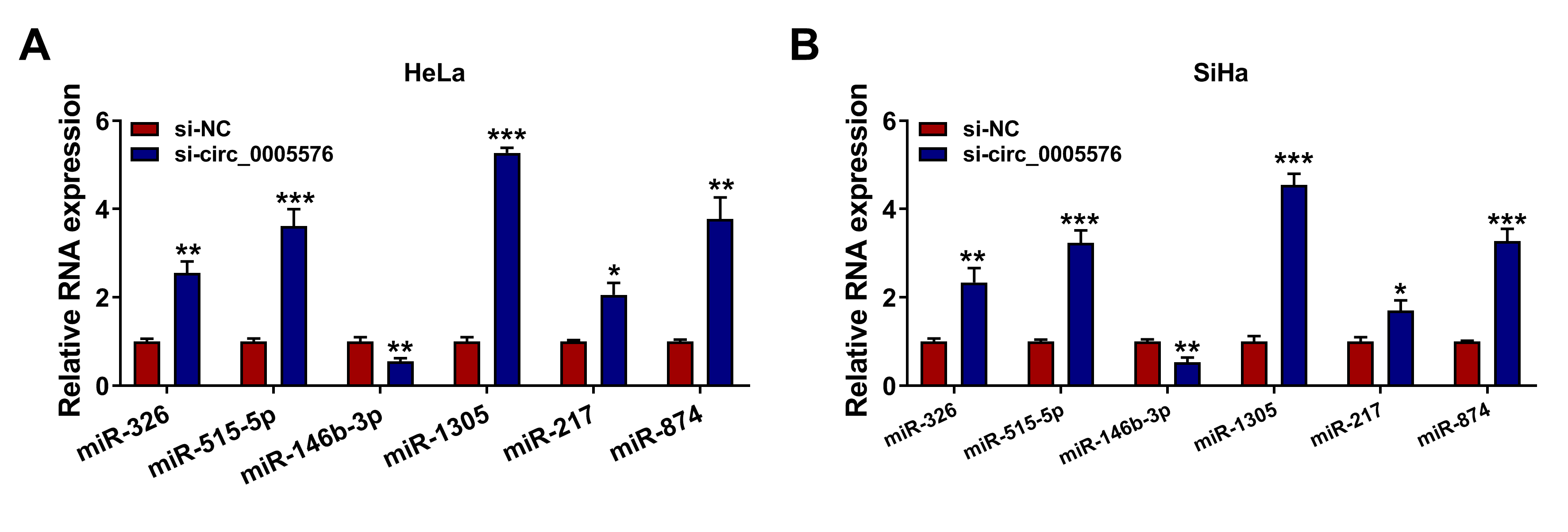

Supplement: Supplementary file 5 — The relative expression of miRNAs in HeLa and SiHa cells after circ_0005576 knockdown. (A and B) The expression levels of different miRNAs in HeLa and SiHa cells transfected with si-circ_0005576 or si-NC were detected by qRT-PCR. *P < 0.05, **P < 0.01, ***P < 0.001.(PNG 103 kb) [file 43032_2022_925_Fig10_ESM.png]

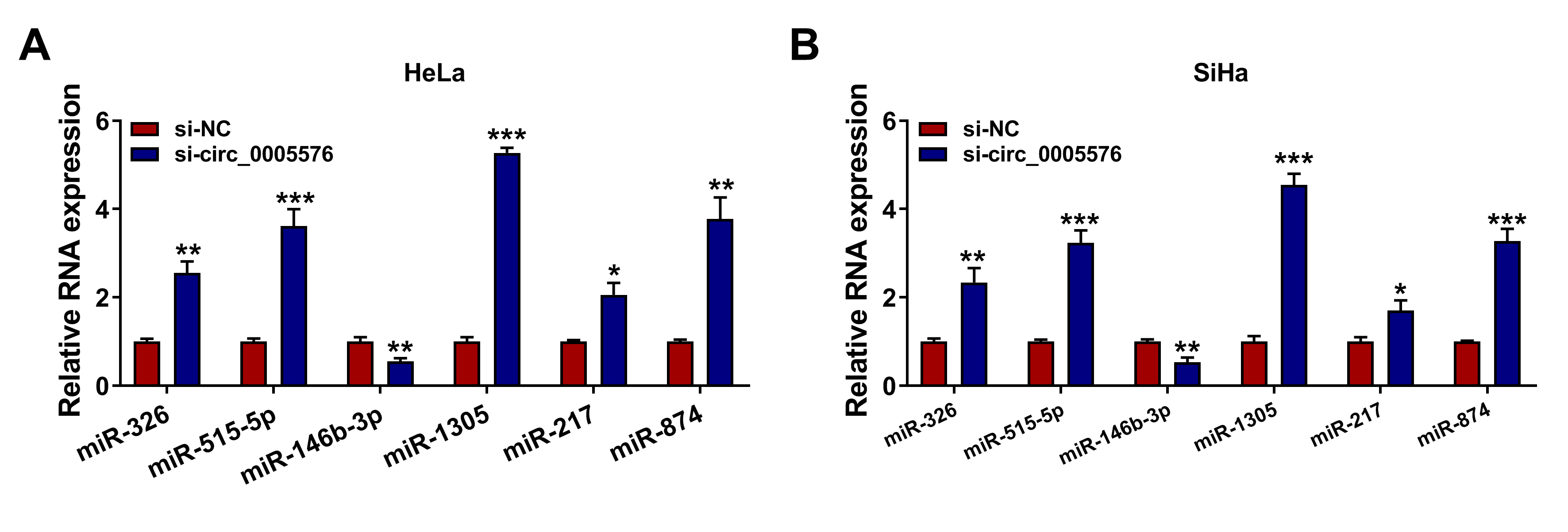

Supplement: Supplementary file 6 — High Resolution (TIF 373 kb) [file 43032_2022_925_MOESM3_ESM.tif]

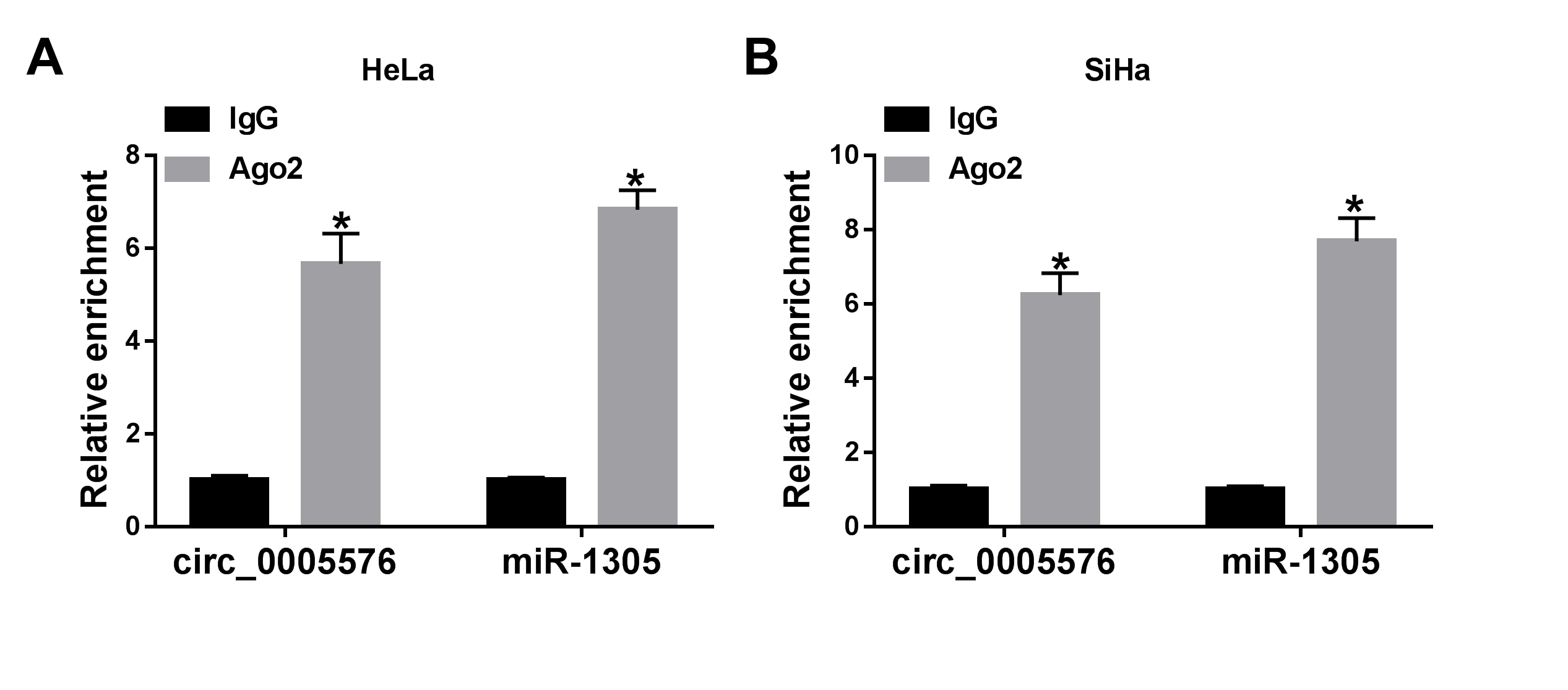

Supplement: Supplementary file 7 — The binding between circ_0005576 and miR-1305 in CC cells. (A and B) RIP assay was used to verify the binding between circ_0005576 and miR-1305 in HeLa and SiHa cells. *P < 0.05. (PNG 46 kb) [file 43032_2022_925_Fig11_ESM.png]

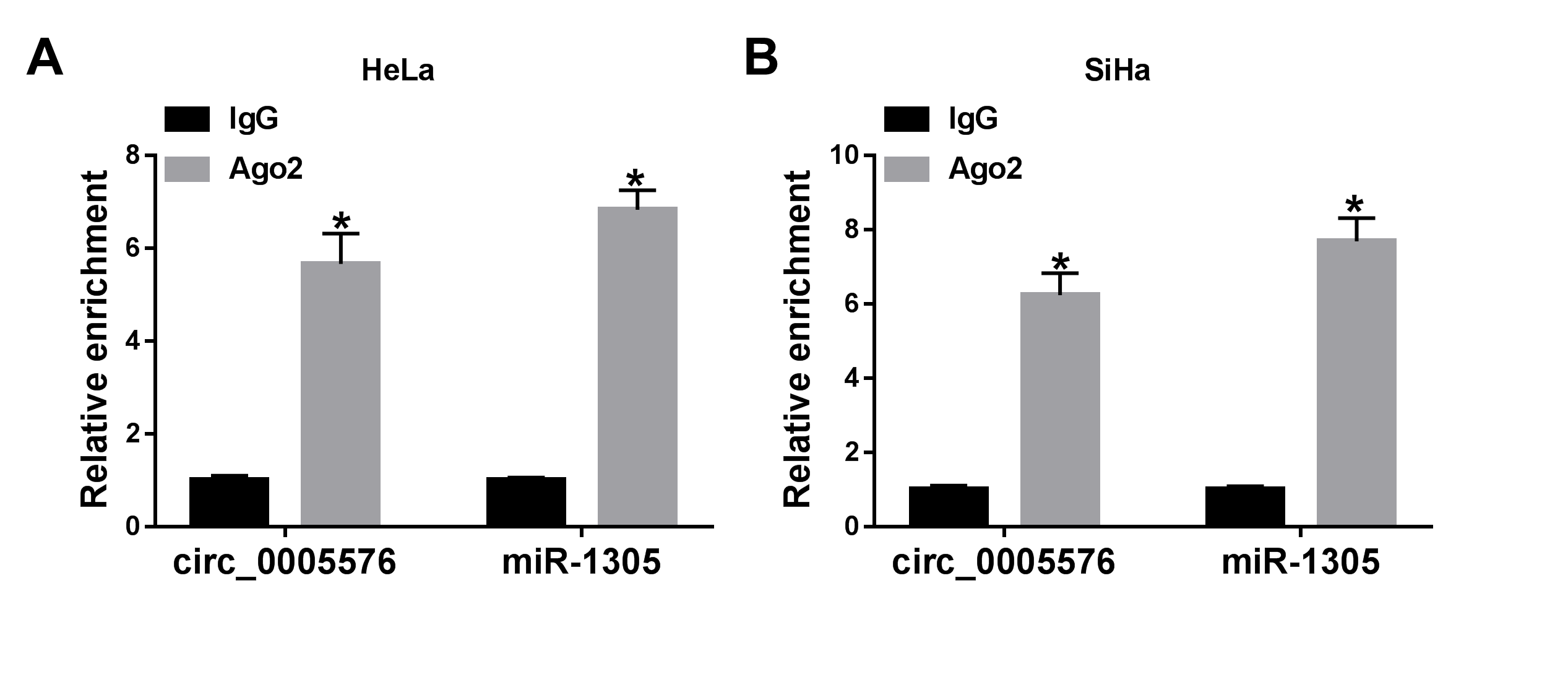

Supplement: Supplementary file 8 — High Resolution (TIF 236 kb) [file 43032_2022_925_MOESM4_ESM.tif]

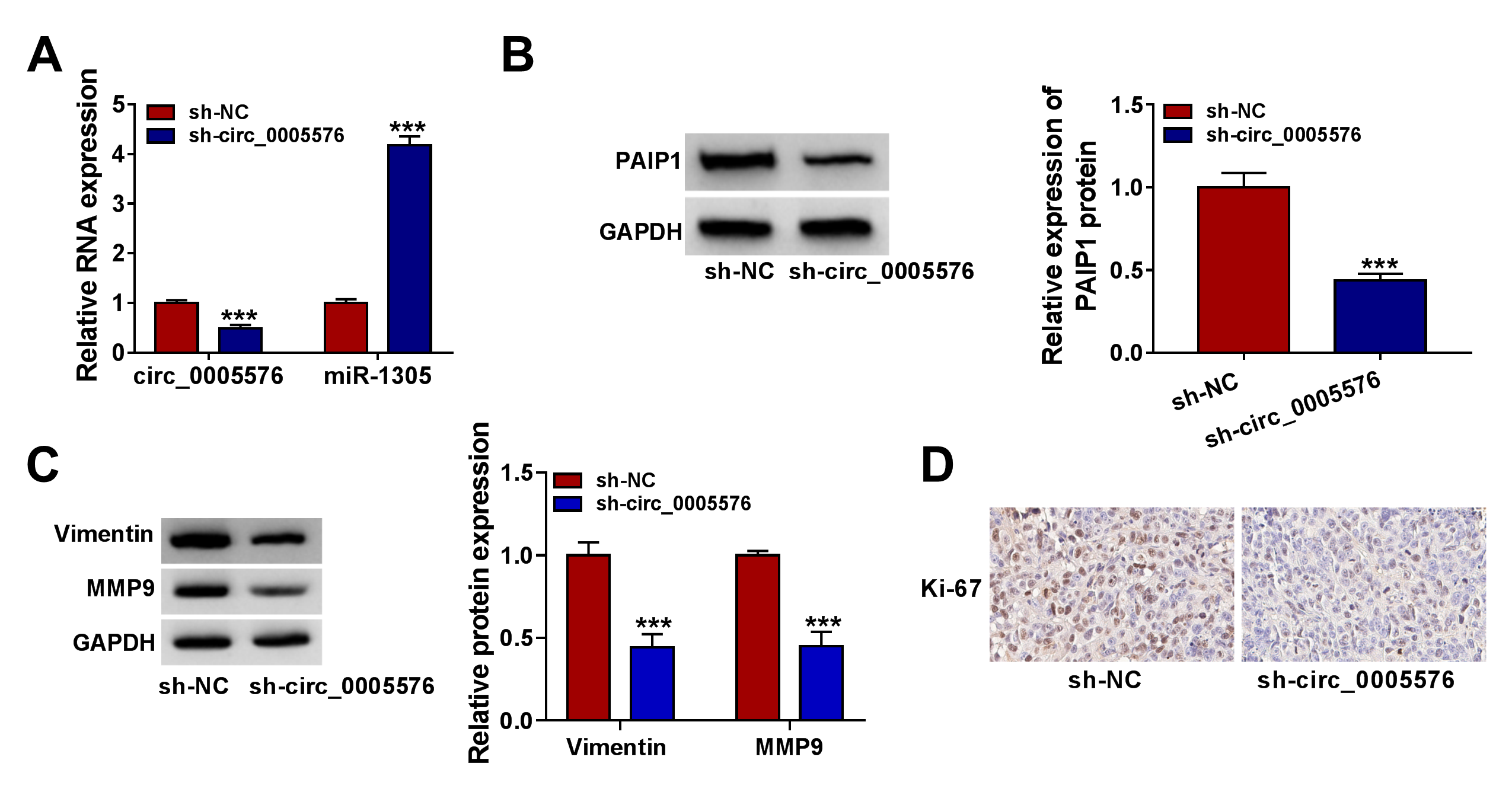

Supplement: Supplementary file 9 — The functional role of circ_0005576 silencing in vivo. (A) The levels of circ_0005576 and miR-1305 were examined by qRT-PCR. (B) The protein level of PAIP1 was detected by western blot. (C) The protein levels of Vimentin and MMP9 were examined by western blot. (D) The Ki-67-positive cells in tumor tissues were detected by IHC analysis. ***P < 0.001. (PNG 615 kb) [file 43032_2022_925_Fig12_ESM.png]

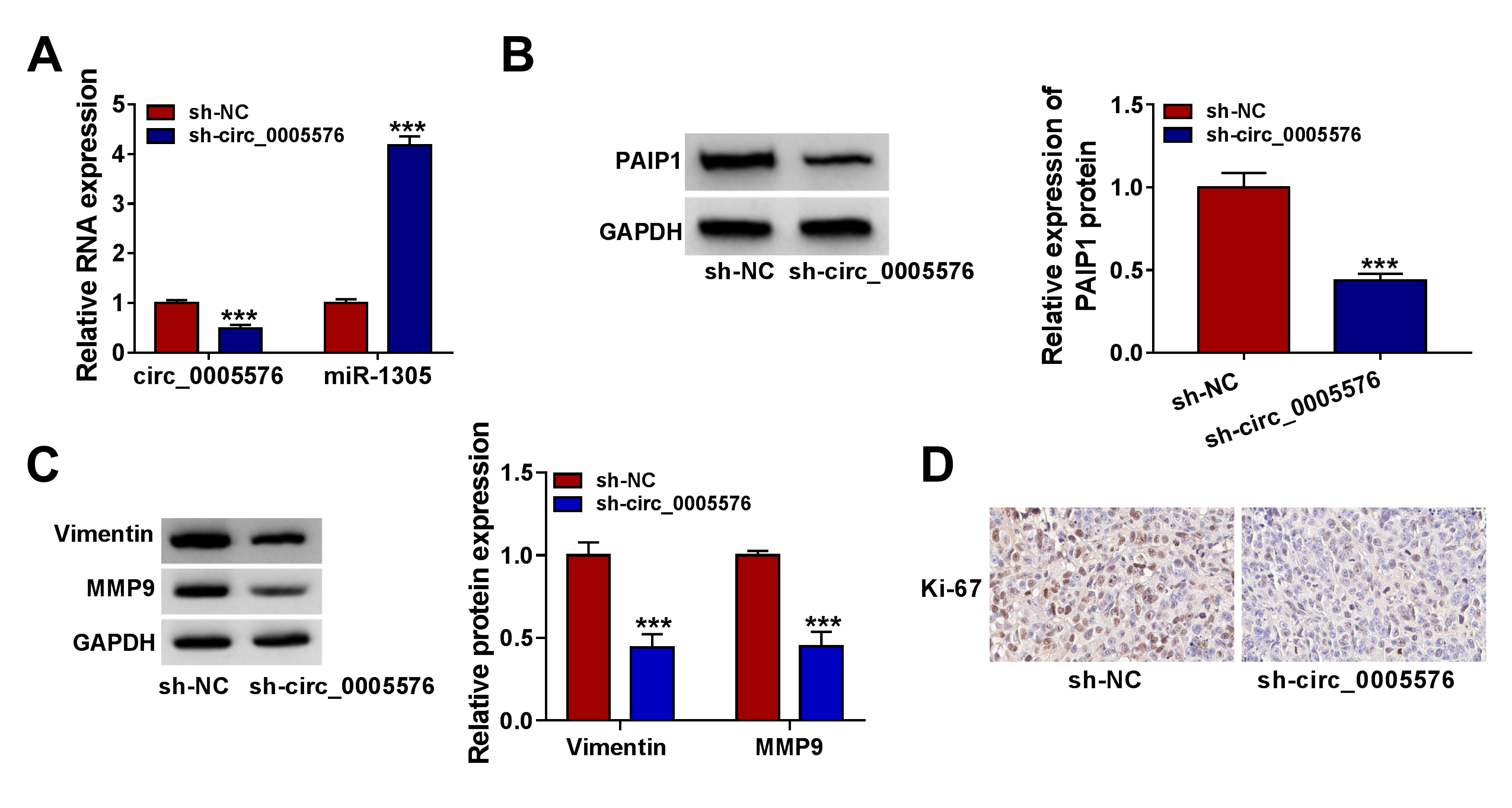

Supplement: Supplementary file 10 — High Resolution (TIF 1037 kb) [file 43032_2022_925_MOESM5_ESM.tif]
